# The impact of lipoprotein lipase deficiency on health-related quality of life: a detailed, structured, qualitative study

**DOI:** 10.1186/s13023-017-0706-1

**Published:** 2017-09-19

**Authors:** Sasi Neelamekam, See Kwok, Rachel Malone, Anthony S. Wierzbicki, Handrean Soran

**Affiliations:** 1 Cardiovascular Trial Unit, Central Manchester University Hospitals NHS Foundation Trust, Manchester, UK; 2 Division of Cardiovascular Sciences, University of Manchester, Manchester, UK; 3 Chiesi Limited, Manchester, UK; 4 Guy’s and St Thomas’ Hospitals, London, UK

**Keywords:** Burden of disease, Chylomicronaemia, Health-related quality of life, Hypertriglyceridaemia, Lipoprotein lipase deficiency, Pancreatitis

## Abstract

**Background:**

Lipoprotein lipase deficiency (LPLD) is an autosomal recessive inherited disorder caused by loss-of-function mutations in genes involved in the lipoprotein lipase pathway. It is characterised by chylomicronaemia, severe hypertriglyceridaemia and an increased risk of recurrent pancreatitis that often requires hospitalisation. This research aimed to improve our understanding of the debilitating impact that LPLD has on the daily lives of patients and their families.

**Methods:**

The research comprised a 2-h interview with the patient and, where possible, a 1-h interview with a family member; a 1-week pre- and post-interview task (written and/or video diary); and a 30–45-min follow-up telephone interview. Feelings and thoughts at each stage of the disease journey were captured on a 0–10 rating scale, while the impact of disease on overall health status was measured via the EuroQoL 5 domains, 3 levels (EQ-5D-3L) questionnaire (descriptive and visual analogue scale).

**Results:**

Of four patients identified, three (two female, one male) were recruited to participate in the study; the male patient did not complete the pre-interview task or consent to a family member interview. Demographics and medical history differed among patients in terms of age at symptom onset, their journey to LPLD diagnosis, treatments, the number of attacks of pancreatitis and lengths of hospitalisations. Health-related quality of life, assessed by the EQ-5D-3L, was poor during acute attacks of pancreatitis but was minimally impacted by their condition at interview. Patients described feeling apprehensive, frightened, anxious, depressed or frustrated during and after hospitalisations; spouses of the two female patients also reported being worried or afraid. LPLD affected many aspects of daily living, including diet; socialising and building relationships; state of mind (fear of another attack of pancreatitis or lack of disease control); college and working life (through absenteeism and consequent financial implications); and being reliant on family and friends for support.

**Conclusions:**

The interviews of the three patients with LPLD highlighted several concerns and emphasised the need for improved education, support, dietary advice and appropriate disease management. Additional support services would ease the fear and uncertainty surrounding attacks of pancreatitis, and would allow for improved treatment during hospitalisations.

**Electronic supplementary material:**

The online version of this article (10.1186/s13023-017-0706-1) contains supplementary material, which is available to authorized users.

## Background

Familial lipoprotein lipase deficiency (LPLD, sometimes known as chylomicronaemia syndrome) is caused by loss-of-function mutations in genes involved in the lipoprotein lipase pathway and is the result of autosomal recessive inheritance [1]. Clinical features include severe hypertriglyceridaemia, chylomicronaemia and an increased risk of recurrent pancreatitis [2, 3]. The estimated prevalence of LPLD is approximately 1 per million population [1, 3], which equates to about 65 people living with LPLD in the UK [4]. The majority of individuals with familial LPLD develop symptoms before 10 years of age, and about 25% of those affected show symptoms before the age of 1 year [1, 3]. Males and females are affected equally [3].

Lipoprotein lipase is the central enzyme responsible for the breakdown of triglyceride-rich lipoproteins known as chylomicrons [5]. In LPLD, clearance of chylomicrons from plasma is impaired, resulting in the accumulation of chylomicrons and therefore, triglycerides in blood. This accumulation, particularly in capillaries, leads to a build-up of triglycerides in adipose and skeletal tissues [2, 6]. Indeed, patients with LPLD can have fasting triglyceride levels 10- to 100-fold greater than normal values (≤1.7 mmol/L) [6]. The ‘chylomicron plugs’ in capillary beds and corresponding high triglyceride levels lead to the signs and symptoms characteristic of the condition, including abdominal pain, which is usually a result of acute pancreatitis, eruptive xanthomata, hepatosplenomegaly, lipaemia retinalis, peripheral neuropathy and cardiopulmonary symptoms [1, 3, 7].

The risk of acute pancreatitis increases with plasma triglyceride levels above 10 mmol/L [8–10]. Pancreatitis associated with LPLD is often recurrent and unpredictable, and can be severe and necrotising [11, 12], with attacks that typically occur at a younger age than with non-hereditary causes of hypertriglyceridaemia [13, 14]. Irrespective of the cause, recurrent episodes of acute pancreatitis may culminate in chronic disease. This can increase the risk of exocrine and endocrine insufficiency resulting from the destruction of pancreatic parenchyma, leading to diabetes [6, 13]. Patients experiencing an attack of pancreatitis often require hospitalisation to control pain and to treat complications, such as multiple organ failure [13]. Severe attacks can be fatal: intensive care unit and in-hospital mortalities of 31% and 42%, respectively, have been reported in the UK, compared with mortality as low as 1% for mild pancreatitis [11, 15]. These attacks may also include a variety of associated medical problems and impaired psychosocial functioning [11, 13, 16, 17].

The mainstay of treatment for LPLD is a very low fat diet, which aims to decrease fasting triglyceride levels to below or close to 10 mmol/L by restricting the amount of dietary fat to 20 g/day or less (or 15% of the total energy intake) [3, 18]. Nevertheless, almost 30% of patients with LPLD develop pancreatitis despite adhering to such a diet [14]. During acute attacks, temporary cessation of food intake with intravenous fluid hydration can be used to stop the production of post-prandially secreted chylomicrons from the gut. In severe acute pancreatitis, which often requires hospitalisation and sometimes intensive care unit admission [19], excess chylomicrons can be removed physically by plasmapheresis or haemodialysis [18].

The symptoms of LPLD, the need to follow a fat-restricted diet and the impaired psychosocial functioning associated with the disease have an impact on health-related quality of life (HRQoL) [7, 20]. Furthermore, a lack of effective therapies for LPLD is likely to add to the burden of disease. This research aims to improve our understanding of the impact that LPLD has on patients and their relatives, and to demonstrate clearly the challenges these people face in daily life. This was achieved through detailed, structured interviews with three patients in the UK and their family members.

## Methods

### Ethical approval

The study (IRAS Project ID: 173627; REC reference number: 16/YH/022; NIHR CRN reference: META 30909) was approved by the Yorkshire and the Humber – Sheffield Research Ethics Committee. Informed consent to participate was obtained from eligible patients and all participating family members; ethical approval was granted for inclusion of a maximum of four patients. According to the protocol and the conditions laid down by the ethics committee, participants confirmed that the case studies were an accurate representation of their interview and were made aware that the anonymised results may be published in peer-reviewed journals. The ethics committee agreed that no further participant consent would be required for publications originating from the anonymised case studies.

### Patients

Patients were identified (screened) from a review of their medical notes at two tertiary lipid clinic databases to confirm that they met the research inclusion criteria. Potential participants were subsequently contacted by their regular LPLD clinician and were invited to join the study to enable three patient case examples to be investigated. All those identified were approached for consent.

Individuals interested in participating in the study were sent patient information sheets and informed consent forms. Those who required additional information or wished to proceed were asked to contact Synergy Healthcare Research, London, UK (the clinical research organisation commissioned by the study sponsor to conduct the research, including all interviews). Patients were informed that their research documentation would only be accessible to Synergy Healthcare Research, and that neither their name nor any other identifiable information would be disclosed outside of this organisation unless explicit consent was obtained. Although patients were not informed of the identity of the study sponsor, they were told that a pharmaceutical company was sponsoring the research.

Patients who were eligible to take part in the study underwent additional screening by Synergy Healthcare Research to ensure that they met the inclusion criteria. Male or female adult patients (aged 18 years or older) with a clinical diagnosis of LPLD confirmed by genetic testing were eligible for study inclusion. Patients also had to have fasting triglyceride levels above 20 mmol/L at the time of screening and a history of acute pancreatitis or abdominal pain consistent with pancreatitis. Patients with secondary causes of hypertriglyceridaemia (e.g., excess alcohol intake or uncontrolled diabetes) were excluded from the study.

### Study design and interviews

The study was based on interviews with the patient and, where agreed, a family member, in addition to diary entries from the patient. Overall, the research comprised five elements: a 2-h face-to-face interview with the patient; where possible, a 1-h face-to-face interview with the patient’s family member (held on the same day as the patient interview); a 1-week pre- and a post-interview task; and a 30–45-min follow-up telephone interview. For the 1-week pre- and post-interview task, patients were requested to complete a written and/or video diary at the end of each day. Participants were asked to spend at least 2–3 min recording the impact that LPLD had on their life on that particular day. They were advised to consider how they felt, any symptoms that they had, how LPLD and/or their symptoms had affected them and their family/friends, the food they had eaten or avoided, effects on general activities of daily living and the impact of their condition at work. The face-to-face interview was the minimum level of participation a patient was required to consent to in order to take part in the study. All other tasks were optional (including the interview with the family member). Patients and family members were offered £40–£80 per task as compensation for their participation.

During the face-to-face interview, time was spent exploring the patient journey from symptom onset, through diagnosis and previous/current treatments. The effect of symptoms or acute episodes of pancreatitis formed a key discussion point. Feelings and thoughts at each stage of the patient journey and during periods of significant symptoms or episodes of acute pancreatitis were captured using showcards with a 0–10 rating scale and a combination of words, colours or faces. The patient was also asked to complete the EuroQoL 5 domains, 3 levels (EQ-5D-3L) questionnaire [21]. This is a standardised instrument for use as a measure of health outcome and provides a simple descriptive profile and a single index value for health status [21]. The EQ-5D-3L employs a descriptive system, in which the patient is required to indicate his/her health state by ticking one of three boxes against the most appropriate statement (1, no problem; 2, some problems; 3, extreme problems) for each of the five dimensions assessed (i.e., mobility, self-care, usual activities [work, study, housework, family and leisure activities], pain/discomfort and anxiety/depression). This questionnaire also uses a visual analogue scale (VAS; score of 100 = best state imaginable; score of 0 = worst state imaginable) [21] to quantify the impact of LPLD on overall health status. In each case, the patient was asked to consider two different scenarios: (i) how they remembered feeling during their most severe acute attack of pancreatitis; and (ii) how they felt at the time of the interview.

### Analysis

Data were analysed descriptively. To obtain overall mean scores in the EQ-5D-3L and the VAS for all three patients, the mean of individual ratings for each patient was calculated for each time point (during the most severe attack compared with at the time of the interview).

## Results

### Patient demographics and medical history

Of four patients identified and screened, three were recruited (two from Manchester and one from London, UK) to participate in the study (patients 1, 2 and 3). One patient did not meet full entry requirements when completing the screening questionnaire because he did not answer any question directly and instead referred all questions to his doctor. Two of the three recruited patients completed the pre-interview diary, and all three completed the face-to-face interview, post-interview diary and follow-up telephone interview. For patients 1 and 3, a family member was also interviewed; however, patient 2, who did not complete the pre-interview diary, did not consent to having a family member interviewed.

Patient demographics and medical history are presented in Table 1. The age at symptom onset and LPLD diagnosis varied among the three patients. The journey to diagnosis for patient 1, the oldest participant, was long and complex. She first developed symptoms at the age of 13 years and was later (at 18 years old) diagnosed with hyperlipidaemia type I/V and acute pancreatitis; however, she did not receive a confirmed diagnosis of LPLD until more than 30 years later. Patient 2 was the youngest of the three patients included in the study. He first developed symptoms when at primary school; he was diagnosed with LPLD as a young child, following the diagnosis of an older sister. Patient 3 had a succession of recurrent severe acute attacks of pancreatitis in her mid-to-late 30s, which occurred over a relatively short period of time; a diagnosis of LPLD was given 3 years later.

**Table 1:** Patient demographics and medical history, based on patients’ recollections of events

	Patient 1	Patient 2	Patient 3
Sex	Female	Male	Female
Age (years)	64	28	42
Age at LPLD symptom onset (years)	13	Primary school age	Early 30s
Age at diagnosis of LPLD (years)	Early 50s (diagnosis of hyperlipidaemia type I/V and acute/chronic pancreatitis at 18 years old)	Young age	Early 40s
Number of acute attacks of pancreatitis requiring hospitalisation	>15^a^	102^b^ (requiring hospitalisation over 5 years)	5 (2 admissions to ITU; 2 to HDU; 1 admission to a ward)
Medications^c^ taken at home for LPLD symptoms	Antox®, black garlic	Tramadol, cyclizine, Deep Heat® patches	Statins, fenofibrate, omega-3 capsules
In-hospital medications/management during attacks of pancreatitis	IV fluids and morphine, slow re-introduction of food	IV fluids, morphine and cyclizine followed by IV paracetamol, nil by mouth for 1–2 days	Analgesic medications, total parenteral nutrition
Comorbidities	PVD, hypertension, IBS	None	Type 2 diabetes
Family members with LPLD	Sister had a child (son) who died from high levels of lipids in the liver (formal diagnosis unknown)	Yes (sister)	Yes (sister)

*HDU* high-dependency unit, *IBS* irritable bowel syndrome, *ITU* intensive therapy unit, *IV* intravenous, *LPLD* lipoprotein lipase deficiency, *PVD* peripheral vascular disease

^a^This number may be underestimated given that patient 1 experienced one attack per month in her late teens and early 20s

^b^This number was estimated by the patient

^c^Pharmacological and alternative

All three patients follow a fat-restricted diet. With the exception of dietary intervention, medications taken at home differed among the three patients (Table 1). Patient 2 was also receiving investigative therapy for his condition.

Patient 3 has two children, who were born between her diagnosis of diabetes and the development of her first symptoms of LPLD. Patient 1 was sterilised in her late teens following the diagnosis of hyperlipidaemia type I/V after being told she should not have children because of her illness. Patient 2 has no children.

All three patients had similar symptoms, including acute pancreatitis, chronic pancreatitis (patients 1 and 2 only), abdominal pain, vomiting and nausea, back pain and plasma lactescence. Patient 1 also had hepatosplenomegaly and new-onset cardiovascular disease, while patient 3 additionally had steatorrhoea. Reported comorbidities were generally cardiometabolic in nature, except for concomitant irritable bowel syndrome, reported by patient 1. Patient 3 also had type 2 diabetes, which was diagnosed in her early 30s.

The number of acute attacks of pancreatitis requiring hospitalisation in these patients also differed (Table 1). Patient 1 estimated that she had had over 15 attacks requiring hospitalisation. However, this number may be underestimated given that she reported experiencing one attack per month in her late teens and early 20s. Patient 2 had the impression that he had had a considerable number of acute attacks requiring hospitalisation (estimated at 102 admissions in total). He reported that attacks increased in severity as he became older. Patient 3 reported that she had had five attacks requiring hospitalisation (one mild, four severe); she was the only patient admitted to the intensive therapy unit (ITU) and high-dependency unit during hospitalisations. The longest periods of hospitalisation were reported to be 15 weeks (3 weeks in a local hospital followed by 12 weeks in a metabolic unit), 5 days and 2 weeks for patients 1, 2 and 3, respectively.

### Impact of LPLD on HRQoL

All three patients remembered that their HRQoL was poor during the worst acute attack of pancreatitis, as assessed by the EQ-5D-3L. However, they confirmed that their HRQoL was minimally impacted by their condition at the time of the interviews. The average health status of all three patients across four of the five domains (mobility, self-care, usual activities and pain/discomfort) of the EQ-5D-3L descriptive system was level 3 during acute attacks and level 1 at the time of the interview. The average health status for anxiety/depression was level 1.7 during acute attacks and level 1 at the time of the interview. When the average health status of all three patients was assessed using the VAS, it was 14.7 during acute attacks and 90.0 at the time of the interview (see Additional file 1 for individual scores).

**Fig. 1:**
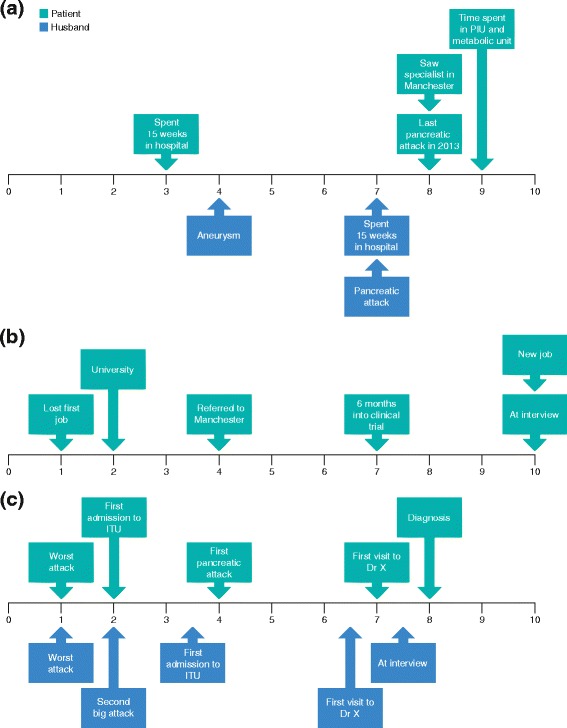
Timeline of feelings: **a** Patient 1 and her husband; **b** patient 2; **c** patient 3 and her husband. Feelings at key points in the patient’s and family member’s disease journey for the three patients were rated using a score of 0 (extremely negative) to 10 (extremely positive). *ITU* intensive therapy unit; *PIU* programmed investigation unit

Figure 1 depicts a timeline of feelings at key points in the disease journey. Patient 1 did not believe that her HRQoL had been affected by LPLD; she has a very positive attitude and reported that she did not allow her illness to control her. She confirmed that she leads a healthy and active lifestyle alongside her spouse. During her teens/early adulthood, when she experienced many acute attacks of pancreatitis, she reported remaining unperturbed, despite believing that she did not have long to live. This patient, however, did describe feeling apprehensive during the 15-week hospitalisation following an attack of pancreatitis in her 30s (Fig. 1a). Her husband is also very pragmatic, but described worrying about a potential attack whenever his wife has a stomach ache or back pain. 

By contrast, patients 2 and 3 reported periods of depression and anxiety (Fig. 1b and c). Patient 2 described a 6-month period of depression following two acute attacks of pancreatitis that occurred during his first job after finishing university:

“*That was the worst where I was just at home with nothing literally … I felt it was my prime time and I was losing it all to this* [the illness] *and I had no answers from anybody.*”

This patient also reported feeling stressed and frustrated during hospitalisations, having to explain to medical staff that his condition was not alcohol related:

“*That was the worst thing, when you felt like death … I used to say straightaway, it’s not alcohol related, I had this from birth. But it never really sank in*.”

Patient 3 provided detailed accounts of her emotions and described being “*frightened, worried and alone*” during her first ITU stay, following a severe attack. In between hospitalisations, patient 3 reported being constantly worried that she would experience another attack, and feeling miserable and frustrated that nobody could do anything to prevent it.

“*I worried every day about it* [having another attack] *and it’s the question of when’s this going to happen again, not if.*”

After her worst attack (which led to ITU admission for 2 weeks), patient 3 was very frightened: “*… most terrified I’ve ever been in my life*”. It was after this period, following discharge, that she reported becoming increasingly anxious and depressed, not only owing to the fear of recurrent pancreatitis episodes and hospitalisations but also because of a lack of control over her illness. Her husband also described feeling “*frightened, concerned and nervous*” following his wife’s worst attack.

### Burden of disease

In addition to the burden of illness experienced by the three patients and their families, LPLD was described as having a substantial psychological impact, driven by fear or the threat of another attack and by the impact that the disease had on daily living. A key area for all three patients was adhering to a strict diet, which they found to be challenging. The dietary interventions placed upon patients with LPLD also result in limitations with respect to the use of convenience foods and eating out owing to the uncertainties of dietary composition of foods. Patient 1 described spending a considerable amount of her daily routine preparing and cooking meals. One of her key concerns surrounded the fear of being unable to control her diet later in life when she might not be able to cook for herself. Patient 2 described having to forego his mother’s cooking, which had led to tensions in the family and having to resort to a “*boring*” and easily digestible diet, such as chicken, tuna and pasta. Importantly, patients stated that they had generally received little useful dietary support from their healthcare professionals.

LPLD also influenced the university and working life of these patients. Patient 2 reported that his illness has affected his education, career, and social and personal life. The increased frequency of pancreatitis and hospitalisations during his time at university meant that he was ill for ~50% of this time; consequently, this probably affected his degree grade. Patients 1 and 2 did not disclose their illness to employers until attacks of pancreatitis resulted in hospitalisations. For all patients, employers have generally been supportive and understanding. However, having LPLD resulted in recurrent work absenteeism – sometimes for extended periods (up to 33 weeks for patient 1), reduced/part-time working hours and, subsequently, financial concerns (particularly for patients 2 and 3).

Patients described having to rely on their families or friends for support during attacks and hospitalisations. However, a lack of understanding of the seriousness and cause of the disease from family members sometimes led to raised anxiety or tension during attacks. For example, patient 3 reported that she had the impression that her parents thought the attacks were self-inflicted because she was not adhering to her diet, while patient 2 described keeping his family “*out-of-the-loop*” to avoid stressful situations. Patient 1 described how, during attacks in her teenage years, her father did not tell her mother of the attacks to avoid worrying her; consequently, the mother was not informed of hospitalisations and patient 1 did not receive any visitors during this stage.

Other issues reported included the need to live in certain locations, as in the case of patient 1 who was advised that diesel fumes could exacerbate her condition; as a result, she lives in a remote area to avoid fumes. Patient 3 faces the challenge of having to decide whether or not to have her children tested for LPLD and the impact that a positive diagnosis would have for them.

### Unmet needs

Table 2 lists key areas of unmet need identified by these three patients. One of the main concerns was the lack of knowledge and understanding of LPLD and pancreatitis by healthcare professionals. Patients and family members reported having to conduct much of their own research into LPLD because they felt that the information they were receiving from their general physicians and other healthcare professionals on the disease was inadequate. In some cases, patients/spouses had to inform medical staff upon hospital admission that they suspected an acute attack of pancreatitis to facilitate appropriate care. In relation to this lack of understanding, patients described how appropriate treatment was often delayed during hospitalisations. As previously mentioned, patients were occasionally perceived to have alcoholism when they presented at hospital with symptoms, causing further distress and delays in treatment. In addition, healthcare professionals often gave insufficient, inconsistent and/or inappropriate dietary advice, making it difficult for patients to adhere to the required diet and thereby control their disease. Lack of patient and family support, and understanding from the wider healthcare community was also highlighted by patients, who reported a high unmet need for emotional support and counselling.

**Table 2:** Unmet needs highlighted by patients with lipoprotein lipase deficiency

Patient 1	Patient 2	Patient 3
• Medical education and support from HCPs • Ensure that medical records are passed to relevant onward care providers	• Medical education and support from HCPs • Early access to disease expert/specialist • Early access to effective and well-tolerated treatments • Advice and emotional support for patients and family members	• Dietary advice • Better understanding of pancreatitis • Prompt diagnosis • HCP support during long gaps between outpatient appointments • Emotional support • Information to aid patient and family disease knowledge/understanding

*HCP* healthcare professional

## Discussion

The case reports presented here give an insight into daily life for patients with LPLD and their family members. The individuals described in this study are currently controlling their condition through a fat-restricted diet, and, in the case of patient 2, with additional investigative therapy. However, the daily challenges they face, as well as the acute attacks of pancreatitis and associated hospitalisations, are an indication of the experiences of many patients with LPLD. It should be remembered that despite adhering to a fat-restricted diet, almost 30% of individuals with LPLD develop pancreatitis [14]. Furthermore, the patients described fear and anxiety associated with attacks of pancreatitis. There is clearly an unmet need for healthcare services to provide support and education for people with this condition.

While the presenting symptoms of LPLD appeared to be relatively consistent among the patients in this study (acute/chronic pancreatitis, abdominal pain, vomiting and nausea, back pain and plasma lactescence), there was undoubtedly much variation in other aspects of the disease profile. The age at symptom onset, number and severity of attacks and comorbidities, treatments and general patient journey to diagnosis were all different, suggesting that there is no ‘typical’ patient with LPLD. As might be expected given such differences, each patient dealt with their disease and attacks of pancreatitis differently, and described a range of emotions, from feeling very positive and pragmatic to experiencing fear, depression and anxiety.

Patient 3’s two children were born between her diagnosis of diabetes and the development of her first LPLD symptoms. There are published reports of hypertriglyceridaemia and attacks of pancreatitis occurring during pregnancy, after which a diagnosis of LPLD has been established [22–24]. This suggests that pregnancy may aggravate the disease in some women, inducing symptoms that may not have occurred previously or that went unnoticed. Healthcare professionals should be aware of the impact of pregnancy on the development of the disease, and investigate the possibility of a diagnosis if symptoms suggestive of LPLD occur in pregnant women.

LPLD has a significant impact on patients’ HRQoL, as demonstrated by results obtained from the EQ-5D-3L tool when assessing four of the five domains (mobility, self-care, usual activities and pain/discomfort). During attacks of pancreatitis, patients gave their HRQoL a low score; in contrast, HRQoL was minimally impacted by their condition at the time of the interviews. The frequency and severity of attacks of pancreatitis resulting in hospitalisations, sometimes for very long periods, affected overall HRQoL. For example, patient 2, who reported experiencing 102 attacks of pancreatitis that required hospitalisation over a 5-year period, scored his health on the EQ-5D-3L VAS during that period as 4/100 (where 0 is the worst state imaginable). The impact of hospitalisations on HRQoL, combined with the uncertainty surrounding the frequency and severity of attacks, is likely to create substantial worry for patients. In particular, this may be the case for patients with young families, who may not have relatives nearby to help and/or who may be concerned about the emotional impact of the hospitalisations on their children. Interestingly, the average health status for anxiety/depression was scored relatively high during acute attacks as well as at the time of the interview (level 1.7 versus level 1, respectively), even though patients 2 and 3 reported that their condition had resulted in periods of depression and anxiety. Despite the worry and stress reported by patients, they generally try to remain positive throughout episodes of hospitalisation. The low VAS score reported during attacks of pancreatitis may be due in part to a reported lack of awareness and limited support from healthcare professionals, resulting in stress and uncertainty in the patients and their family members. LPLD also had a substantial effect on patients’ careers; irregular working hours necessitated by the condition are likely to result in financial concerns, as described by the patients in this study.

There is a requirement for improved general education on LPLD among healthcare professionals and a need for increased awareness of symptoms among hospital staff, particularly in those initially encountered by patients during attacks (e.g., accident and emergency department staff) to prevent delays in treatment. To this end, it may be useful to issue diagnosed patients with an identifying medical card or bracelet [25], which would explain their diagnosis and describe emergency treatment steps to healthcare professionals to prevent delays in treatment when they are hospitalised.

There is an unmet need for appropriate and consistent dietary advice, as well as education and sources of information on LPLD for patients and family members. Education of healthcare professionals should be improved to ensure that appropriate diets are followed during hospitalisations and to allow patients to better understand and control their condition through diet at home. There is also an unmet need for appropriate counselling, emotional support services and support groups. Patients and their family members consistently described a high level of fear surrounding attacks of pancreatitis. Provision of such services and materials offering sources of information would help to ease family tensions and anxiety, and enable patients to understand their disease better.

This assessment of HRQoL and the burden of disease in LPLD provides insight into the key unmet needs that patients face. The results reported here reflect those of a recent publication based on a comprehensive online research survey of 60 patients with LPLD [20]. Similar to the current work, Davidson et al. reported that most patients experience a lengthy journey to diagnosis, and substantially reduced HRQoL as a result of their illness [20]. LPLD was also reported to influence patients’ career choices and employment status. Furthermore, patients described high levels of anxiety, fear and worry surrounding attacks of pancreatitis. The results reported by Davidson et al. support and strengthen those uncovered in this study, emphasizing the burden of disease and unmet need that patients with LPLD face.

A limitation of this study is the low number of participants. This is an unavoidable limitation resulting from the rarity of the disease and the low numbers of patients with LPLD in the UK, and may limit the interpretations presented. Future research should focus on examining the impact of LPLD on HRQoL in patients from other countries to determine whether the issues raised here are applicable on a broader scale. Some of the key unmet needs identified in this study may not apply in other regions; findings from other countries may therefore provide insights into how to address these issues.

## Conclusions

The interviews with these three patients with LPLD highlighted several concerns, emphasising the need for education, support and appropriate management. Additional support services, such as those described above, would ease the fear and uncertainty surrounding attacks of pancreatitis, and would facilitate improved treatment during hospitalisations.

## Additional file

Additional file 1: HRQoL during the worst attack of pancreatitis and at the interview, assessed by EQ-5D-3L VAS. Score of 0 = worst state imaginable; score of 100 = best state imaginable. *EQ-5D-3L* EuroQoL 5 domains, 3 levels; *HRQoL* health-related quality of life; *VAS* visual analogue scale. (PDF 838 kb)

## Abbreviations

EQ-5D-3L: EuroQoL 5 domains, 3 levels
HRQoL: Health-related quality of life
ITU: Intensive therapy unit
LPLD: Lipoprotein lipase deficiency
VAS: Visual analogue scale
