# Supplementary figures and images for: The impact of lipoprotein lipase deficiency on health-related quality of life: a detailed, structured, qualitative study
Source: Orphanet J Rare Dis. 2017 Sep 19;12:156. doi: 10.1186/s13023-017-0706-1 (PMC5606084; doi:10.1186/s13023-017-0706-1)

Patient 1

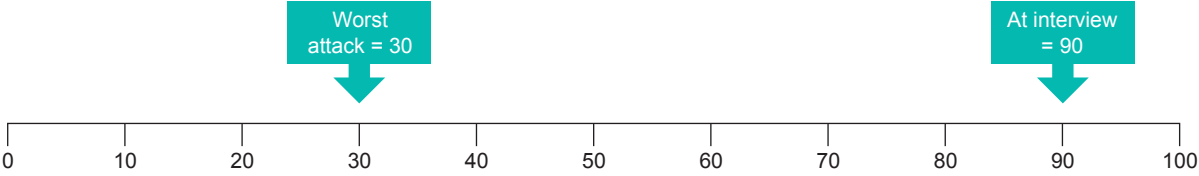

Patient 2

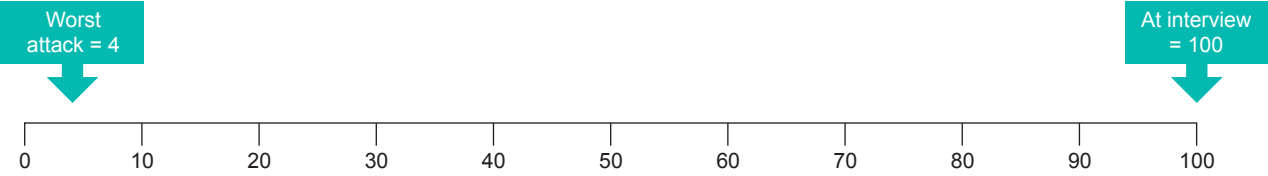

Patient 3

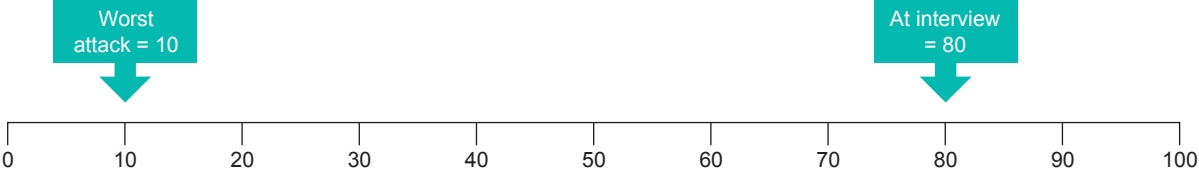

Supplement: Additional file 1: — HRQoL during the worst attack of pancreatitis and at the interview, assessed by EQ-5D-3L VAS. Score of 0 = worst state imaginable; score of 100 = best state imaginable. EQ-5D-3L EuroQoL 5 domains, 3 levels; HRQoL health-related quality of life; VAS visual analogue scale. (PDF 838 kb) [file 13023_2017_706_MOESM1_ESM.pdf]
